# Learning curves and long-term outcome of simulation-based thoracentesis training for medical students

**DOI:** 10.1186/1472-6920-11-39

**Published:** 2011-06-22

**Authors:** Guanchao Jiang, Hong Chen, Shan Wang, Qinghuan Zhou, Xiao Li, Kezhong Chen, Xizhao Sui

**Affiliations:** 1 Department of Thoracic Surgery, Peking University People's Hospital, No 11, Xizhimen South Street, Beijing 100044, China; 2 Department of Cardiology, Peking University People's Hospital, No 11, Xizhimen South Street, Beijing 100044, China; 3 Department of General Surgery, Peking University People's Hospital, No 11, Xizhimen South Street, Beijing 100044, China; 4 Department of Education, Peking University People's Hospital, No 11, Xizhimen South Street, Beijing 100044, China

## Abstract

**Background:**

Simulation-based medical education has been widely used in medical skills training; however, the effectiveness and long-term outcome of simulation-based training in thoracentesis requires further investigation. The purpose of this study was to assess the learning curve of simulation-based thoracentesis training, study skills retention and transfer of knowledge to a clinical setting following simulation-based education intervention in thoracentesis procedures.

**Methods:**

Fifty-two medical students were enrolled in this study. Each participant performed five supervised trials on the simulator. Participant's performance was assessed by performance score (PS), procedure time (PT), and participant's confidence (PC). Learning curves for each variable were generated. Long-term outcome of the training was measured by the retesting and clinical performance evaluation 6 months and 1 year, respectively, after initial training on the simulator.

**Results:**

Significant improvements in PS, PT, and PC were noted among the first 3 to 4 test trials (p < 0.05). A plateau for PS, PT, and PC in the learning curves occurred in trial 4. Retesting 6 months after training yielded similar scores to trial 5 (p > 0.05). Clinical competency in thoracentesis was improved in participants who received simulation training relative to that of first year medical residents without such experience (p < 0.05).

**Conclusions:**

This study demonstrates that simulation-based thoracentesis training can significantly improve an individual's performance. The saturation of learning from the simulator can be achieved after four practice sessions. Simulation-based training can assist in long-term retention of skills and can be partially transferred to clinical practice.

## Background

Thoracentesis is a common procedure that is important for diagnosing and treating pleural effusion. A recent report relayed that most internal medicine residents felt uncomfortable performing the procedure [1]. Potential reasons for discomfort during thoracentesis could be due to the increased risk of life-threatening complications such as pneumothorax and hemothorax that can occur, particularly when thoracentesis is performed by physicians-in-training [1]. Although performing thoracentesis on patients is the most valid method to meet a physician-in-training's training requirement for clinical competency, this approach is labor-intensive, time-consuming, and carries the risk of harm to patients. One potential substitute for direct training, simulation, has been gaining popularity in medical education over the past decade [2-4]. Specialties and procedures such as carotid angiography [5], anesthesiology [6], emergency medicine [7,8], and laparoscopic surgery [9] have led the way in using simulation modalities to teach and test the clinical skills of physicians-in-training. Not only does simulation save faculty time, it is also readily available at any time to reproduce a wide variety of clinical conditions and situations on-demand [3]. Moreover, simulation can circumvent a myriad of ethical obstacles (e.g., pelvic examinations). More importantly, when mistakes occur, trainees can learn to recognize and correct them in a forgiving environment, without fear of punishment or harm to the patients [3,4,10,11].

One report notes that simulation-based thoracentesis practice dramatically improves residents' skills in thoracentesis [12]; however, numerous questions remain pertaining to the learning curve and long-term skill retention for medical students who learn thoracentesis using a simulator. It is also important to learn to what extent simulation-based training can be transferred to actual clinical skill. All these issues are very important for creating a successful training program. The purpose of this study was to analyze the learning curve and skill retention of simulation-based training for thoracentesis.

## Methods

### Subjects and Simulator

From 2007 to 2009, 52 medical students in their fifth year of medical school at Peking University People's Hospital were enrolled in the study. A background questionnaire was administered and identified that no subjects had prior experience in performing thoracentesis on either a simulator or a patient. All students were asked to perform thoracentesis on the simulator to construct a learning curve of simulation-based thoracentesis for medical students. To evaluate the long-term effectiveness of simulation-based training, study participants were followed-up. Their performance of thoracentesis on the simulator (i.e., a retest) and real patients (i.e., clinical competency assessment) were assessed 6 months and 1 year, respectively, following the initial simulation-based thoracentesis. Thirty-two residents who graduated from other medical school without previous simulation-based training were enrolled in this study as a control group for clinical competency assessment.

In total, 10 evaluators were used in this study for assessing test trials, retesting, and clinical competency assessment. These evaluators included senior residents (n = 5), staff (n = 3), and full time teachers (i.e., simulation educators) in the simulation center (n = 2). All of the evaluators were expert in thoracentesis on both the simulator and patients. Nonetheless, all evaluators received the same training regarding assessing the performance of the trainees to improve the reproducibility of the assessment. An inter-rater reliability test was used to confirm the consistency among these 10 trained evaluators.

The thoracentesis task trainer used in this study was an anatomical chest simulator with a realistic replaceable soft skin, palpable ribs, scapula, and intercostal spaces, which allowed the trainee to practice percussion whilst determining the puncture site. This simulator also had a refillable reservoir from which fluid could be withdrawn by needles during the thoracentesis.

### Procedures and Measurements

This study was conducted in the Peking University People's Hospital Simulation Center, and was reviewed and approved by the Peking University People's Hospital Review Board. After signed informed consent and completed background questionnaires were received, all study participants attended a 30 minute didactic training session on indications, risks and complications, procedural technique, post-procedure interpretation, and a step-by-step demonstration of thoracentesis on the simulator. All participants were required to pass a multiple choice written examination prior to practicing thoracentesis on the simulators.

Three performance measurements were conducted: performance score (PS); procedure time (PT); and participant's confidence (PC). PS assessed correct performance of thoracentesis according to a 21-item checklist (Table 1), which was developed for the thoracentesis procedure using relevant sources [13,14]. Each skill or action was scored as either 1 (i.e., performed correctly without any error) or 0 (i.e., performed incorrectly). PT was recorded by an evaluator according to the time spent by the participant on each test trial. PC was reported by the trainee according to a 5-point Likert scale, which was designed to reflect self-confidence in performance.

**Table 1:** Checklist for Procedures of Thoracentesis

1. Explain procedure to the patient, obtain a written informed consent;
2. Measure blood pressure;
3. Positioning the patient;
4. Recognizing the anatomic landmarks;
5. Percussion combine with CRX to determine the puncture site;
6. Wear hat, mask and gloves;
7. Checking equipment components;
8. Applying sterile technique;
9. Anesthetize the skin first;
10. Anesthetize deeper to pleura (just over the upper border of the rib);
11. Aspirate while advancing, and watch for color change;
12. Notice the depth of the needle advanced;
13. Change to the pleural puncture needle, advance carefully;
14. Aspirate while advancing until the color is the same as before;
15. Flexible the needle with nipper, connect the needle with drainage bag;
16. Aspirate the fluid slowly;
17. Let patient hold breath when withdrawing the needle;
18. Cover the puncture site with sterilized materials;
19. Send pleura fluid to lab for different examination;
20. Measure blood pressure again and have a auscultation;
21. Talk with patient to find any possible complications

To construct a graph of proficiency comparing the trainees and experts and to determine the mastery level with thoracentesis on the simulator, each of the 10 evaluators performed three thoracenteses using the simulator. The evaluators' performances were evaluated according to the 21-items checklist. The PS for senior residents, staff, and full time teachers were 19.2 ± 0.8, 18.9 ± 0.6, and 19.7 ± 0.5, respectively. Accordingly, the learning objectives of the simulation-based thoracentesis training for medical students were defined as achievement of the level of a senior resident's performance. That is, the training would be discontinued on achievement of a PS not less than 19. This means that > 90% of the procedures listed in the 21-items checklist were correct. Although some students reached this level in only a few trials, each participant was asked to perform at least five thoracentesis on the simulator so that a learning curve could be generated.

Each participant's performance of thoracentesis on the simulator was assessed by one of the ten evaluators using the measurements of PS, PT and PC. Learning curves for each variable were generated. Evaluators did not interrupt the participants while they were performing the procedures. Participants were given overall procedural feedback on their performance according to the PS checklist at the end of each test trial by the evaluator so that they could improve their performance in the next trial. Participants could attend the practice session at their convenience by either performing five thoracenteses either on the same day or different days (but within a 2 week period).

Finally, trainees were followed-up and requested to perform a thoracentesis on the simulator 6 months after the initial training period. In addition, the trainees' first thoracentesis on a clinical patient, performed approximately one year after training, was also evaluated. The same group of evaluators (excluding the full time teachers) performed these assessments.

### Statistical analysis

All statistical analyses were performed with SPSS version 15.0. Differences between performances on test trials were examined for significance with analysis of variance (ANOVA) for repeated measures. Specific contrasts between mean scores for different trials were compared for statistical significance with Scheffe F-tests for controlled multiple comparisons.

## Results

### Characteristics of Participants

**Figure 1:**
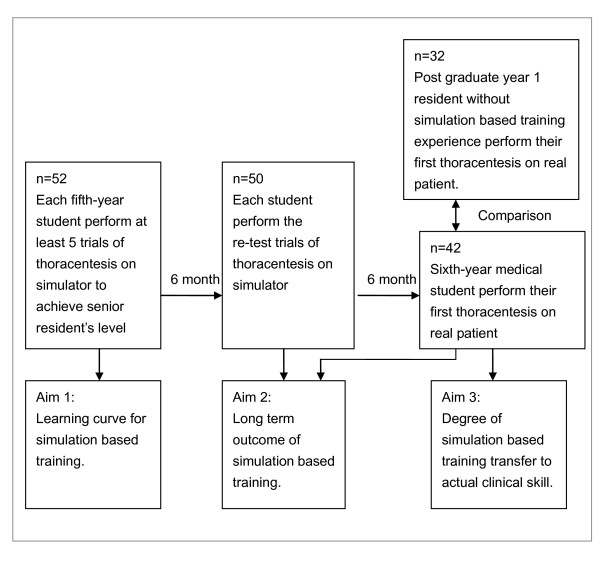
**Flow chart showing how participants completed each of the 6 trials of thoracentesis on simulator and the actual patient care**.

Fifty-two fifth year medical students (24 females) were enrolled in this study. None of the included students had any prior experience performing thoracentesis. Among these participants, 50 were retested on the simulator 6 months after the initial training and 42 performed the thoracentesis on clinical patients 1 year after training (i.e., while in their sixth year of medical school). An additional 32 postgraduate first year residents who were not trained at the Peking University People's Hospital were also enrolled in this study to serve as the control group. Demographic details of the trainees who performed thoracentesis on clinical patients 1 year after training on the simulator have been summarized in Table 2. A flow chart showing how participants completed the retest and thoracentesis on a patient has been provided in Figure 1.

**Table 2:** Comparison of personal characteristics actual clinical skills between the residents with and without simulation based training experience

	participants with training experience	participants without training experience	P value
Total	42	32	
Training year in hospital, (%)			
Postgraduate first-year residents	0	32(100%)	
Sixth-year medical students	42(100%)	0	
Gender, (%)			0.990
Female	17(40%)	13(41%)	
Male	25(60%)	19(59%)	
Self-reported prior procedures, (n)			
Simulation based	6.5	0	
Real patient based	0	0	
witnessed thoracentesis	4.2 ± 0.7	4.4 ± 1.1	0.282
Score of pre-test *	47.1 ± 1.3	46.9 ± 2.5	0.624
Clinical Competency Assessment			
Performance Score	19.2 ± 0.9	18.7 ± 1.1	0.034
Performance Time	10.4 ± 1.0	12.5 ± 1.4	< 0.001
Performance Confidence	4.4 ± 0.5	3.6 ± 0.6	< 0.001

* Pre-test, it is a multiple choice test on the knowledge about thoracentesis.

### Test for Inter-rater Reliability

To test the inter-rater reliability, two trainees' first and second thoracentesis on the simulator were assessed by all 10 of the evaluators at the same time. The inter-rater reliability for PS was 0.989 (range, 0.986-0.991). This result demonstrated excellent inter-rater reliability in scoring the trainees' performances (Table 3).

**Table 3:** Test for inter-rater reliability of the 21-items checklist

	*E1	E2	E3	E4	E5	E6	E7	E8	E9	E10
*P1-1^st^	16	14	14	15	14	15	14	15	15	14

P1-2^nd^	18	18	18	18	17	18	18	18	18	16

P2-1^st^	16	15	15	16	14	14	15	15	15	15

P2-2^nd^	17	18	18	18	16	17	18	18	18	17

* E means evaluator; P means participant; P1-1^st ^, 1^st ^test of participant 1

### Learning Curve

**Figure 2:**
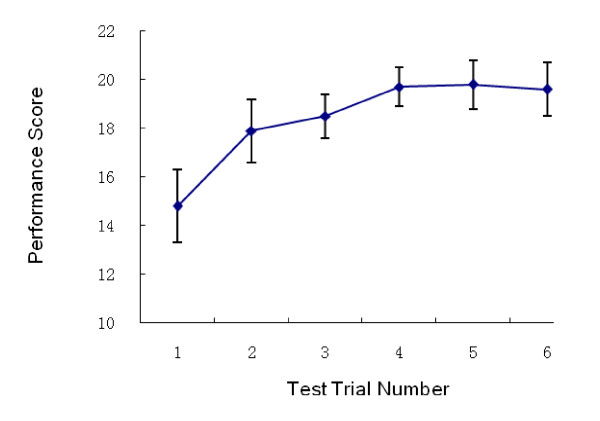
**Performance Score on 5 test trials and the retest (Trial 6)**.

**Figure 3:**
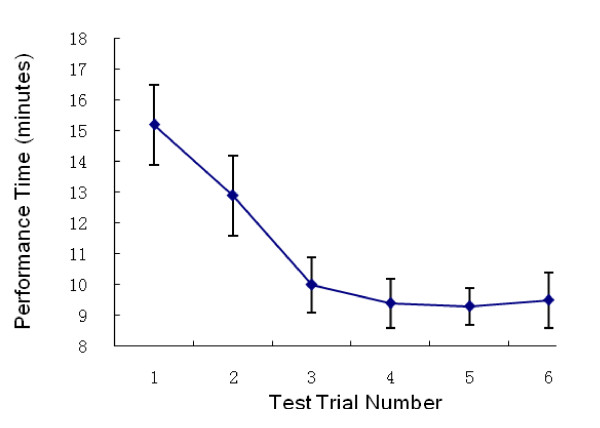
**Performance Time on 5 test trials and the retest (Trial 6)**.

**Figure 4:**
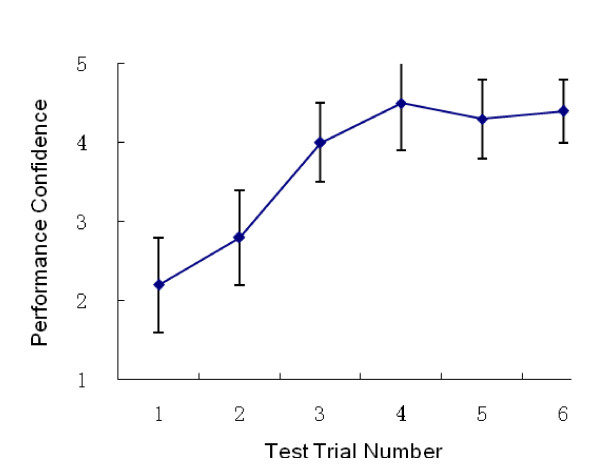
**Performance Confidence on 5 test trials and the retest (Trial 6)**.

The trainees' assessment scores were summarized. Figures 1, 2 and 3 show the PS, PT and PC through the five trials on the simulator and the retest performed 6 months after initial training. The overall PS increased significantly between trials 1 and 5. The sharpest increase in PS occurred between trials 1 and 2. The curve reached a plateau at trial 4. Similarly, PT decreased at trial 4, with an overall significant reduction in PT over the course of the five trials. An increase in PC was noted, which also reached a plateau at trial 4. Most of the trainees agreed that four trials were necessary and sufficient for trainees to become confident performing this procedure. Considerable variability in PS, PT and PC, as evidenced by the large standard deviation scores in trials 2 and 3 compared to trials 4 and 5 was noted. A decrease in the performance variability (Figures 2, 3 and 4) suggested a consistent improvement in operating performance among the trainees who received simulation-based training.

### Long-term Outcome

Long-term outcome of the simulation-based training was determined by retesting on the simulator and by the trainees' clinical performance evaluations. Fifty (96%) trainees were retested using simulation-based thoracentesis six months after the initial training. No significant difference between the trial 5 scores and the retest scores were noted (Figures 2, 3 and 4). Forty-two (80%) of the trainees had the opportunity to perform a thoracentesis on a clinical patient one year after training. The performances of the trainees' first thoracentesis on patients were assessed by the senior residents or staff, using the same scoring tool. The trainees' performances were compared with the performances of the 32 first year residents who graduated from other medical schools without previous experience using simulation-based thoracentesis training. The postgraduate training in thoracentesis of these 32 first year residents included didactic training and observation (Table 2). All 74 participants wrote and passed a multiple choice written examination prior to being permitted to perform the thoracentesis on a patient. The study participants who received simulation-based thoracentesis training had better performances on their first thoracentesis than residents without such experience. There was no significant difference in demographic data and pre-test scores between the two groups (Table 2).

## Discussion

The aim of this study was to document the learning curve of simulation-based thoracentesis training. In addition, this study also evaluated the long-term outcome and the effectiveness of simulator training on clinical performance. All of these issues were deemed important in evaluating simulation-based thoracentesis training and designing successful training programs.

This study demonstrates that inexperienced medical students are able to master the necessary skills to competently perform thoracentesis via simulation-based trials. To assess performance, 21 skill-related measures, such as choosing aspiration site and needle aspiration, were assessed [13,14]. The reliability and face validity of all 21 skills have been confirmed in clinical skills examination in the Peking University People's Hospital and have been well documented in the literature [13,14]. Although performing a procedure faster does not necessarily translate to improved performance, performing a procedure in a reasonable period of time could be a considered a crude assessment of the technical performance of the operator. Performance confidence, meaning "comfort" when performing a procedure, has also been proposed as a marker of competence in several studies [1,15,16]. Thus, when performing a given procedure, residents with a low comfort level are potentially unlikely to be competent.

The slope of the learning curve demonstrates a change in learning with each successive attempt. At some points over repeated attempts, the change in learning may be so small that the slope flattens or nearly flattens. At this point, the plateau, the full effect of learning is achieved. Over-training may not gain more positive results. In this study, the authors found that the curves of PS, PT, and PC reached their plateaus at trial 4. Score variability of each trial reflects the consistency of the performances and is an important parameter of skill acquisition. In this study, a great deal of performance variability was initially noted by the high standard deviation in the first two or three trials in all three curves. The degree of variability decreased considerably in trials 4 and 5. This decrease in operator variability in the setting of improved performance suggests that acquisition and firm establishment of proper procedural technique were probably reached in trial 4.

It has been reported that simulation-based training benefits clinical practices in carotid angiography[17] and surgical skills [18]. This study suggests that simulation-based thoracentesis training could also be transferred to an actual clinical setting. These results show that the sixth year medical students with simulation training experience have better performance in a clinical setting than first year residents without such experience. It should be noted that the structure of the undergraduate medical curriculum is diverse in China. In the authors' hospital, the medical curriculum was of 8 years duration and medical students begin to study in the hospital after the third year. The curriculum for sixth year medical students in the authors' hospital is equivalent to that for first year residents who graduated from a medical school with a 5 year program. Therefore, the two groups assessed in the clinical setting were comparable in their overall clinical exposure and experience.

It was challenging to obtain permission from the patients and their relatives to have the thoracentesis performed by a resident or a medical student despite the fact that all thoracenteses were supervised by a clinical physician. This was the reason why the study lasted over 2 years and the average time between the retest and the clinical thoracentesis was 6 months (range, 3-16 months). This long duration could have influenced the results. The authors also noted that after the two groups had performed thoracentesis on three or more patients, there was no difference in performance between the sixth year students and first year residents (data not shown). This may reflect that the transfer of simulation training to clinical skill is primarily in the first two clinical practices.

It is worth mentioning that several important steps involved in thoracentesis cannot be trained and tested using the simulator, such as how to communicate with the patients, and how to observe the patients' reaction. The simulation-based training provides a safe and relaxed environment for the students to grasp the procedures, but operator confidence still needs to be obtained whilst performing the procedure in a clinical setting. Thus, simulation-based training can not be used as a substitute for clinical practice [19].

## Conclusions

This study demonstrates that simulation-based thoracentesis training can significantly improve an individual's skill and knowledge. This type of training assists with long-term skill retention and can be partially transferred to clinical practice. Likely, the full effect of learning from a simulator can be achieved by performing the procedure four times. Over-training may not result in further increase in competence or confidence. The authors conclude that simulation-based thoracentesis training should be a key element for future medical education.

## Competing interests

The authors declare that they have no competing interests.

## Authors' contributions

All authors participated in the design of the study, review of the data, and manuscript preparation. HC, GJ, and XL were principally responsible for data analysis. All authors reviewed and approved the final manuscript.

## Pre-publication history

The pre-publication history for this paper can be accessed here:

http://www.biomedcentral.com/1472-6920/11/39/prepub
